# High Neuraxial Blockade in a Parturient After Epidural Placement: A Quality Improvement Simulation

**DOI:** 10.7759/cureus.77712

**Published:** 2025-01-20

**Authors:** Samir Patel, Noah D Le, John Carter, Nick Melott, Andrew Kalnow, Camille D Hawkins, Alexander Luong, Matthias Franzen

**Affiliations:** 1 Anesthesiology and Perioperative Medicine, OhioHealth Doctors Hospital, Columbus, USA; 2 Anesthesiology, Ohio University Heritage College of Osteopathic Medicine, Dublin, USA; 3 Anesthesiology, Des Moines University, Des Moines, USA; 4 Emergency Medicine, OhioHealth Doctors Hospital, Columbus, USA; 5 Anesthesiology, OhioHealth Doctors Hospital, Columbus, USA

**Keywords:** academic anesthesiology, anesthesiology resident, epidural anaesthesia, epidural injections, neuraxial block, obstetric anesthesiology, pregnant females, quality improvement projects, simulation medicine, teaching by simulation

## Abstract

Background

Epidural anesthesia is a commonly used procedure in obstetrics for managing labor pain, with a potential complication of high neuraxial blockade. Proficiency in performing epidural or spinal anesthesia and promptly recognizing and managing high neuraxial blockade are critical components of anesthesiology residency training.

Methods

A simulated case was designed involving a 21-year-old female in labor receiving epidural anesthesia. After the administration of local anesthetic, she develops symptoms of nausea, hypotension, and respiratory distress, progressing to high neuraxial blockade. The simulation environment included a labor and delivery suite setup, an epidural cart, airway supplies, a Laerdal SimMan 3G PLUS mannequin (Laerdal Medical, Stavanger, Norway), and an M43B Kyoto Kagaku Lumbar Puncture Simulator II (Kyoto Kagaku, Kyoto, Japan). Post-simulation, participants completed surveys via REDCap (Vanderbilt University, Nashville, USA) to assess their comfort levels in recognizing and managing high neuraxial blockades.

Results

Nine participants, including anesthesiology residents (PGY1-4) and student registered nurse anesthetists (SRNAs), completed the pilot simulation. Survey feedback was collected to evaluate the simulation’s impact on participants' learning and confidence.

Discussion

Participants provided positive feedback, highlighting the simulation’s value in their training and its realism. Suggestions for improvement included using an actual labor and delivery bed to better mimic real-world challenges. A limitation of the study is the small sample size, additional sessions with future cohorts will help further assess the simulation’s efficacy. Plans are in place to incorporate this simulation annually into the anesthesiology residency curriculum at the institution.

Conclusion

Simulation-based training in anesthesiology residency programs enables learners to practice managing complex and high-stakes scenarios in a controlled environment. This simulation effectively reinforced critical skills, including endotracheal intubation, epidural catheter placement, and the management of a hemodynamically unstable pregnant patient. Furthermore, it provided valuable experience in recognizing and treating high neuraxial blockade, a serious but rare complication of epidural anesthesia.

## Introduction

Epidural anesthesia, a form of neuraxial anesthesia, involves the placement of a catheter in the epidural space to administer a combination of local anesthetics and opioids, resulting in sensory blockade and analgesia. Epidurals are commonly used in obstetrics for labor and delivery analgesia in both vaginal and cesarean section deliveries [1]. While generally safe, epidural anesthesia is associated with adverse effects such as hypotension, headache, urinary retention, non-reassuring fetal heart tones without neonatal adverse outcomes, and prolonged second-stage labor. There are more serious complications that can arise from the administration of neuraxial anesthesia, some of which include respiratory arrest and an unrecognized spinal catheter [2]. Among these, the most common serious complication is the high neuraxial blockade, or “high spinal,” characterized by unintended cephalad spread of the anesthetic, leading to hypotension, bradycardia, and respiratory depression [2].

Correct technique and placement of an epidural catheter is an essential skill that an anesthesiology resident must develop during their training. Furthermore, being able to quickly recognize and treat the symptoms of high neuraxial blockade in a pregnant patient undergoing labor and delivery is part of the American Society of Anesthesiologists (ASA) Guidelines for Obstetric Anesthesia [3] and should be a component of an anesthesiology residency program’s curriculum. As high neuraxial blockade is the most common serious complication of neuraxial anesthesia in obstetrics, anesthesiologists need to be able to quickly recognize the signs and symptoms and treat them appropriately.

This simulation involves a healthy 21-year-old female at 41 weeks gestation presenting for labor induction. Following epidural catheter placement for labor analgesia, the patient develops symptoms of nausea and hypotension, progressing to high neuraxial blockade with bradycardia and respiratory depression. This simulation is intended for residents of an anesthesiology residency program but could be a useful exercise for fellows and attending anesthesiologists as well. Additionally, Certified Registered Nurse Anesthetists (CRNAs) and SRNAs could benefit from taking part in the simulation. The simulation is intended to be run by a senior resident or faculty; however, its design should ensure that it may be run by proper simulation center staff assuming all necessary equipment and confederates.

The learning objectives of this simulation align with several Accreditation Council for Graduate Medical Education (ACGME) Program Requirements for Graduate Medical Education in Anesthesiology [4], including:

IV.B.1.b).(1).(c).(ii): Competence in anesthetic care for cesarean section patients.

IV.B.1.b).(1).(c).(vii): Competence in the use of epidural anesthesia for perioperative analgesia.

IV.B.1.b).(1).(c).(ix): Competence in spinal anesthetic care for cesarean section patients.

IV.C.14: Participation in at least one simulated clinical experience annually.

This simulation also addresses milestones in both general anesthesiology and obstetric anesthesiology [5,6], such as Medical Knowledge (MK) 1 and 2, and Patient Care (PC) 1, 3, and 10.

There are few peer-reviewed simulations published on MedEdPortal regarding neuraxial anesthesia and high neuraxial blockade. A search using the term “neuraxial anesthesia” performed on June 27, 2022, yielded three such cases, and only one of which is related to a parturient and discusses the administration of neuraxial anesthesia to a patient with Tetralogy of Fallot [7]. Another search on the same date using the term “epidural” yielded 14 additional cases, only one of which involved a patient in labor who experienced intrapartum maternal cardiac arrest upon administration of an epidural catheter for analgesia due to amniotic fluid embolism [8]. Of note, a PubMed search yielded one study published in *Anesthesiology and Pain Medicine *in February 2020 that discusses the effects of a simulation study of high neuraxial blockade in a patient who received epidural analgesia on pre- and post-simulation scored responses [9]. This study, which was conducted in Japan, emphasizes the effect that the simulation had on its learners' correct response rate rather than the design and implementation of the simulation and its protocols. To date, there is no peer-reviewed simulation within MedEdPortal that discusses the management of a parturient who develops high neuraxial blockade upon the administration of epidural anesthesia. This simulation fills this gap by providing comprehensive tools to incorporate the exercise into residency training and educate anesthesia learners.

## Materials and methods

Development

This simulation was initially piloted with two anesthesiology residents in a one-hour session and will now be implemented annually for each residency class. Participants should possess foundational knowledge and competence in obstetric anesthesia, neuraxial techniques, and airway management. 

Equipment/environment

This simulation required access to a SimMan 3G PLUS mannequin (Laerdal Medical, Stavanger, Norway) or a similar high-fidelity mannequin. Additionally, access to an M43B Lumbar Puncture Simulator II (Kyoto Kagaku, Kyoto, Japan) or a similar spinal/epidural anesthesia training device is needed. Other necessary equipment included an epidural cart with a sterile epidural kit, sterile gloves, monitoring equipment to meet ASA standard monitoring guidelines, and airway equipment (laryngoscope, endotracheal tube, bag-valve mask, etc.). Simulation of medications and fluids was achieved through labeled syringes, vials, and bags. If those materials are not available, learners can verbalize to the facilitators when they are administering medication or fluids. The placement of the epidural catheter was performed as a sterile procedure.

The simulation environment mirrored a labor and delivery suite, with appropriate moulage and props to replicate the setting. If available, an actual labor and delivery bed should be used to enhance realism. Participants and actors were dressed in standard hospital attire, including scrubs, surgical caps, and masks.

Personnel

The simulation required two anesthesia faculty members and a simulation technician. One faculty member facilitated the session and voiced the mannequin’s responses, while the other observed learner actions and adjusted patient vital signs as needed. Of note, if there are touchscreen vital monitors available, the learners and actors should be instructed prior to the simulation that they will be responsible for adjusting the settings of these devices (e.g., changing the time between blood pressure cycling). Ideally, the simulation is performed with two anesthesia residents. One resident takes the role of “junior learner” and, in addition to aiding the “senior learner,” works toward the educational objectives of discussing the risks and benefits of neuraxial anesthesia, obtaining informed consent, and proper patient assessment for the placement of an epidural catheter. The other anesthesia resident should take the role of “senior learner” and work toward the educational objectives of proper sterile technique and competency in placing an epidural catheter, identifying the signs and symptoms of high neuraxial blockade, and demonstrating technical ability to perform emergent endotracheal intubation while teaching/guiding the “junior learner” and collaborating with other specialties (e.g., alerting OB/GYN) in the treatment of a critical, hemodynamically unstable patient. If possible, an actual OB nurse would play their role in this simulation, but if one is not available then an actor is necessary to fulfill this role and help the learners.

Implementation

The simulation began with a pre-brief session in a pre-brief/debrief room with the learners in which they were presented background information on the case including patient history, as well as instructions as to what parts of the simulation they would need to perform versus act out, demonstrate, or verbalize. Learners then transitioned to the simulated labor and delivery suite to initiate the patient encounter.

The simulated case is presented with instructions for use in its entirety in the Simulation Case Template (Appendix A in the Appendices section). Should the learners request more information about the patient (lab findings), those can be found in the Supplemental Materials (Appendix B). The Critical Actions Checklist (Appendix C) for the simulation, which is used to objectively evaluate the knowledge/technical performance of the learners and indicates the steps of an “ideal” scenario, is included as well. Upon completion of the simulation, the learners completed a debriefing session (Appendix D) with the attending anesthesiologists.

Debriefing

We recommend that facilitators use the Promoting Excellence and Reflective Learning in Simulation (PEARLS) approach to debriefing [10]. Learners were given two minutes post-simulation for reflection, followed by a guided discussion about their overall impressions and step-by-step analysis of the case. Additionally, the learners are asked what they think the patient was presenting with (high neuraxial blockade in this case). Afterwards, a two-pronged approach for further analysis could be utilized by the facilitators to increase the educational efficacy of the simulation: 1) +/Δ method to identify areas the learners felt went well versus what they felt could be improved or changed regarding both their performance and the administration of the simulation - 1a. “What did you feel went well during the case?”, 1b. “What are some aspects you liked about the simulation?”, 1c. “What is something you would change about the simulation?”; 2) Advocacy/Inquiry method to address the educational objectives (Appendix A) and critical action checklist (Appendix C) - 2a. “I noticed that you gave glycopyrrolate instead of atropine during the case. Can you tell me what you were thinking when you made that decision?”

Once the educational objectives and individual performances have been addressed, the facilitators should then use the debriefing materials to guide a discussion of key learning points (Appendix D) from the case.

Assessment

Evaluation of the learners is performed throughout the simulation by one of the facilitators following along with the critical actions checklist (Appendix C) as the learners progress through the case. This checklist measures the decisions made by the learners with the “ideal” decisions in clinical management of this case. The simulation has several branching points with triggers that will prompt the facilitators to change the status of the patient should the learners proceed incorrectly. A post-simulation survey assessed participants’ feedback on the session’s educational value and its execution.

## Results

The pilot simulation was run on June 28, 2022, at the simulation lab at Doctors Hospital in Columbus, Ohio. The environment was designed to replicate a labor and delivery suite, complete with essential equipment for epidural catheter placement, a high-fidelity mannequin, and an epidural trainer. The simulation was facilitated by a chief anesthesiology resident, two attending anesthesiologists, and the on-site simulation director. Two anesthesiology residents participated in the simulation: a PGY-1 (junior learner) and a CA-1 (senior learner). Additionally, a medical student played the standardized participant role of an obstetric nurse.

The session began with a pre-briefing, during which learners were provided with background information on the case (Table 1) and instructions regarding the tasks to perform versus those to verbalize or act out (Table 2). Following the pre-briefing, the learners proceeded to the simulation room to initiate the patient encounter.

**Table 1:** Initial Presentation HEENT: head, eyes, ears, nose, throat; GU: genitourinary; N/A: not applicable

Initial Presentation	Findings/Details		
Initial vital signs	Temperature - 36 deg C, Heart Rate - 87, Blood Pressure - 115/84, Respiratory Rate - 14, Oxygen Saturation - 99% on room air, Weight - 55kg, Glasgow Coma Scale - 15 (E4 V5 M6)		
Overall Setting and Appearance	The patient is alert and oriented, in apparent distress, with contractions every three minutes. The learners are in a simulated labor and delivery suite on the OBGYN floor. A female mannequin with the outward appearance of being pregnant is used. A spinal/epidural trainer is located on the patient’s back. The mannequin head includes an intubation trainer if possible; if not, the intubation trainer can be separate. The patient is on a monitor with vitals displayed; monitors will include pulse oximetry (QRS volume set to 0) and a blood pressure cuff running every 15 minutes unless otherwise requested.		
Standardized Participants (and their roles in the room at case start)	In this simulation, the standardized participant may be played by one of the educators (attendings), medical students, or nurses. The standardized participant will play the role of the OB nurse and can help the learner only if asked and will follow the prompts. For questions or tasks asked of the nurse that are unprompted, they may accommodate the learner with a reasonable answer. If any item or medication is requested, the standardized participant will leave the room for 30 seconds and return with the item/medication.		
History of Present Illness	Provided prior to the start of the simulation: The patient is a 21-year-old G1P0 at 40 weeks who presents to the OBGYN floor due to contractions. Records demonstrate an uncomplicated pregnancy. Provided by educators/facilitators upon request: The patient has a 20 gauge IV in the right hand. The patient received a 2L bolus of lactated ringers and bicitra.		
Past Medical/Surgical History	Migraines, no known drug allergies, no surgical history		
Medications	N/A		
Physical Examination			
General	Uncomfortable appearing gravid female. Mallampati class III, normal dentition, mouth opening >3 finger breadths		
HEENT	Normocephalic, atraumatic		
Neck	N/A		
Lungs	Lungs clear to auscultation bilaterally with no wheezes/rhonchi/rales		
Cardiovascular	Regular rate and rhythm with no murmurs		
Abdomen	Appropriate for parturient female		
Neurological	+2 Deep tendon reflexes throughout		
Skin	N/A		
GU	N/A		
Psychiatric	N/A		

**Table 2:** Scenario States, Modifiers, and Triggers NSR: Normal Sinus Rhythm; HR: Heart Rate; BP: Blood Pressure; RR: Respiratory Rate; O2SAT: Oxygen Saturation; T: Temperature; GCS: Glasgow Coma Scale; q3min: Every 3 Minutes; q15min: Every 15 Minutes; OB: Obstetrics; ETT: Endotracheal Tube; FiO2: Fraction of Inspired Oxygen; NC: Nasal Cannula.

Patient State/Vitals	Patient Status	Learner Actions, Modifiers & Triggers to Move to Next State		Facilitator Notes
1. Baseline State Rhythm: NSR HR: 87 BP: 115/84 RR: -- O_2_SAT: 99% T: -- GCS: --	Alert and oriented. Mildly distressed. Contractions every 3 minutes	Expected Learner Actions ☐ Perform a complete assessment of the patient including physical exam, airway exam, and review of labs ☐ Explain the risks and benefits of epidural placement ☐ Check monitors 🡪 BP q3min, HR audible ☐ Position the patient for epidural placement ☐ Demonstrate sterile technique ☐ Demonstrate technical proficiency in placing an epidural ☐ Assess the patient after epidural placement	Modifiers- *Changes to patient condition based on learner action* - BP will be set at q15 mins by default. Learner must request it to be more frequent Triggers- *For progression to next state* * * - Test dose for epidural given. Learner will continue to hook epidural to pump. When pump is started, patient will state that their legs feel weird. Progress to scenario 2	Learner will perform assessment and obtain informed consent from the standardized participant. After this is performed, standardized participant will take the role of the OB nurse. After test dose is given, the learner will listen/ask about heart rate of the patient. The learner must ask about the state of the patient. The patient will state that their legs are feeling “weird”. If asked to elaborate, it will be described as a “tingle” and are “heavy”.
2. Rhythm: Sinus Bradycardia HR: 58 BP: 85/47 RR: -- O_2_SAT: 99% T: -- GCS: --	N/A	Expected Learner Actions ☐ Discontinue epidural infusion ☐ Bolus dose of Ephedrine (5-10 mg) or Phenylephrine (50-100 mcg) ☐ Begin fluid bolus	Modifiers: - After 2 minutes, patient will state that they are lightheaded. - At this time, BP will be 85/47. - If repeat BP is requested, it will be 82/44. Triggers - Pressors given and fluid bolus ordered. Wait 3 minutes unless learner requests a new BP reading. Progress to scenario 3 - If neither are performed, after 60 seconds, progress to scenario 3 with patient complaining that it is difficult to breathe.	At this point, the patient will begin to exhibit bradycardia and hypotension. Patient will be “lightheaded” if asked by learner.
3. Rhythm: Sinus Bradycardia HR: 47 BP: 78/39 RR: -- O_2_SAT: continuously dropping until intervention T: 36^o^C GCS: --	N/A	Expected Learner Actions ☐ Call for help; Notify OBGYN team; Notify Anesthesiology team ☐ Start epinephrine bolus for BP and HR ☐ Obtain Ambu bag, oxygen source ☐ Prepare for intubation; laryngoscope, ETT with stylet and syringe, +/- medications ☐ +/- Sedate patient in preparation for intubation	Modifiers - If request for OBGYN team is made, confederate/”nurse” will leave room for 15 seconds and return stating that the OBGYN team has been notified. - If Anesthesiology team notified, one extra person will be made available to the learner after 45 seconds. - If nasal cannula, simple O2 mask, or nonrebreather requested, confederate will leave for 30 seconds and will return with requested item. - If Ambu bag requested, confederate will leave for 30 seconds and will return with Ambu bag. Pressors/HR Medications - If atropine is given, HR increases to 60 and BP increases to 82/43 after 15 seconds. - If 10 mcg epinephrine is given, BP will increase to 102/54 and HR will increase to 62 after 10 seconds. Repeat bolus -> 123/74 and HR to 96 after 10 seconds - No response to ephedrine or phenylephrine. Vasopressin not available Triggers - If O2 sats drop below 50%, State overhead that OBGYN present for emergent bedside cesarean section (for persistent fetal bradycardia). End simulation. Preparation for intubation - If propofol/fentanyl is given to the patient for sedation, progress to scenario 4. - If etomidate/versed/no sedation is given AND no epinephrine given, progress to scenario 4. - If etomidate/versed/no sedation is given AND epinephrine given, progress to scenario 5.	Patient will complain that it is difficult to breathe and her arms feel weak. No further responses from patient. Oxygen sats will progressively (at a rate of 10%/45 seconds) decrease until supplemental oxygen is started. -If NC/simple mask/nonrebreather mask is used, desaturation rate -> 10%/60 seconds. -If Ambu bag is used, O2 sats will increase at a rate of 10%/30 seconds as long as they are being bagged. If Ambu bag is stopped, O2 sats decrease at 10%/45 seconds Hypotension will not be responsive to any vasopressors other than epinephrine. Bolus doses of 10mcg should be used.
4. Rhythm: Sinus Bradycardia HR: 42 BP: 52/36 RR: -- O_2_SAT: from previous T: 36^o^C GCS: --	N/A	Expected Learner Actions ☐ Intubate patient ☐ Treat BP with epinephrine in appropriate dosages ☐ Prepare patient for transport to OR	Modifiers - If atropine is given, HR increases to 60 and BP increases to 82/43 after 15 seconds. - If 10 mcg epinephrine is given, BP will increase to 102/54 and HR will increase to 62 after 10 seconds. Repeat bolus -> 123/74 and HR to 96 after 10 seconds - No response to ephedrine or phenylephrine. Vasopressin not available Triggers - Patient intubated, Ambu bag attached, and epinephrine bolus given. -If OBGYN team previously notified, overhead that they want to go for emergency cesarean section and to prepare for transport to OR. End simulation -If OBGYN team not notified, confederate/”nurse” will leave to notify OBGYN team. Overhead that they want to go for emergency cesarean section and to prepare for transport to OR. End simulation	- For intubation, a 6.5 ETT or smaller must be used. Mannequin with upper airway edema. - If intubation is performed and Ambu bag is attached and being used appropriately, oxygen sats will increase to 99%. - If intubation is performed and Ambu bag is NOT present, continue desaturation at a rate of 10%/45 seconds - If O2 sats drop below 50%, State overhead that OBGYN present for emergent bedside cesarean section (for persistent fetal bradycardia). End simulation.
5. Rhythm: NSR HR: 65 BP: 105/60 RR: -- O_2_SAT: continuously dropping until intervention T: 36^o^C GCS: --	N/A	Expected Learner Actions ☐ Intubate patient ☐ Prepare patient for transport to OR	Modifiers - N/A Triggers - Patient intubated and Ambu bag attached. -If OBGYN team previously notified, overhead that they want to go for emergency cesarean section and to prepare for transport to OR. End simulation -If OBGYN team not notified, confederate/”nurse” will leave to notify OBGYN team. Overhead that they want to go for emergency cesarean section and to prepare for transport to OR. End simulation	- For intubation, a 6.5 ETT or smaller must be used. Mannequin with upper airway edema. - If intubation is performed and FiO2 is 100%, oxygen sats will increase to 99%. - If intubation is performed and Ambu bag is NOT present, continue desaturation at a rate of 10%/45 seconds - If O2 sats drop below 50%, State overhead that OBGYN present for emergent bedside cesarean section (for persistent fetal bradycardia). End simulation.

The junior learner began by obtaining informed consent for the epidural procedure. This involved discussing the associated risks and benefits with the simulated patient, with the agreement confirmed via loudspeaker by one of the simulation administrators. Both learners then prepared the necessary materials for the procedure on the epidural cart, transitioning from the mannequin to the epidural trainer.

The senior learner assumed the lead role for the procedure. After explaining proper patient positioning to the simulated patient, they prepared and placed the epidural catheter while narrating the steps. Once the catheter was verbalized to be connected to the medication pump and running, the simulated patient reported feeling unwell and exhibited hypotension.

The learners recognized the signs of high neuraxial blockade early on and proceeded to shut off and disconnect the epidural pump. Upon doing so, they began to treat the patient’s symptoms with appropriate vasopressors and ensuring adequate fluid administration. When that did not improve the patient’s condition and they became unresponsive, the learners alerted the OB/GYN team, sedated the patient, and secured their airway via endotracheal intubation. With the airway secure and the Ambu bag attached, the learners gave epinephrine, and the simulation ended with the OB/GYN team (administrators) saying over the loudspeaker that they had arrived for an emergency C-section.

Upon completion of the simulation, the learners underwent a debrief session with the educators/facilitators. In that debrief, they discussed their initial thoughts and reactions to the simulation. Additionally, the facilitators went through the debriefing materials with the learners and reviewed vital information related to the management of this case. The ability of the learners to adequately manage this case was assessed using the critical action checklist (Appendix C). After the conclusion of the debriefing session, the learners completed a survey to determine how relevant they felt the simulation was to their level of training as well as how competent and confident they felt about the management of the case.

**Table 3:** Learner Anticipated Mistakes

Aniticipated Management Mistakes	Expected Learner Actions/Corrections
1. Not identifying signs of high spinal early on	Learners should identify the patient’s complaints, as well as hypotension refractory to ephedrine/phenylephrine, as early signs of a high spinal. Progression to bradycardia and rapid oxygen desaturation should trigger appropriate actions from learners, including calling for the OBGYN team, administering epinephrine, and preparing for intubation of the patient.
2. Not pausing/stopping epidural infusion during hypotension	Upon recognition of hypotension in the patient, the learner should halt the administration of the epidural medication. Further administration could worsen the patient’s hypotension or cause progression to a total neuraxial blockade.
3. Not having emergency medications readily available	Learners should be prepared with readily available epinephrine.
4. Not having necessary equipment ready for intubation	Learners should be prepared with the necessary medications and equipment needed to safely intubate the patient. In this case, it is important to consider the selection of induction medication in a hemodynamically unstable patient. As propofol is associated with decreases in blood pressure, etomidate would be a more appropriate choice for induction.
5. Not having an Ambu bag readily available	Learners should have an Ambu bag readily available to perform manual bag-and-mask ventilation in their resuscitation attempt.
6. Poor communication with the OBGYN team especially in an emergent situation	Communication with the OBGYN team is paramount in an emergent situation, and learners should adequately describe the patient’s hemodynamic status. Additionally, the urgency of the cesarean section in terms of time should be clearly communicated to the OBGYN team.

## Discussion

Epidural anesthesia is a cornerstone of obstetric analgesia, widely used to manage the significant pain associated with labor. The process of labor is extremely painful for the parturient, in part due to cervical dilation, uterine contractions, and perineal stretching. The patient’s perception of this pain, as well as other factors, may lead them to request pain management in the form of epidural anesthesia. If the patient requires a cesarean delivery, a spinal block or epidural is preferred according to ASA guidelines [11] because the baby is exposed to the lowest amount of medication and the mother can still actively participate in the baby’s birth.

Despite its benefits, epidural anesthesia carries the risk of complications, including high neuraxial block, urinary retention, abnormal fetal heart tones, and strong uterine contractions. Unintentional intrathecal injection can result in a high neuraxial block or “high spinal,” characterized by bradycardia, respiratory insufficiency, and hypotension. The initial sign of hypotension may present as nausea, while other initial signs of a high spinal may include numbness/weakness in the upper extremity, dyspnea, and difficulty with phonation. Rapid diagnosis and treatment of a high neuraxial block are critical to the survival of the parturient and fetus, and as such, this case is a valuable resource for an anesthesiology trainee. There is not currently a case simulation with all the necessary components to be implemented across a wide variety of institutions, and as such, this simulation is novel and helps fill a gap in the simulation curriculum for anesthesiology programs.

The residents who underwent the pilot simulation reported positive feedback both in the debriefing session and the post-simulation survey, and they felt it was a valuable experience for their education and training. The learners also made comments on how realistic the simulation felt to them, as well as areas for improvement.

One limitation of this pilot simulation was the small sample size, as it was conducted with only two residents. Ongoing implementation of the simulation on an annual basis will allow for a more robust evaluation of its efficacy and broader applicability. Another potential area for improvement is the simulated environment. Learners suggested using an actual labor and delivery bed rather than a standard hospital bed, as the former’s bulkier design would enhance realism and better simulate the challenges of navigating such equipment during the procedure.

Additionally, variability in debriefing techniques among facilitators may influence the educational impact of the simulation. Standardizing the debrief process could enhance consistency and effectiveness. Facilitators may benefit from pre-simulation meetings to discuss key debriefing points, ensure alignment on critical action checklists, and agree on the flow of the debriefing session. Moreover, giving learners the opportunity to discuss their experiences among themselves before engaging in guided discussions with facilitators may foster deeper reflection and promote critical analysis of their performance.

This simulation will be incorporated into the annual training curriculum for all anesthesiology residents at the Doctors Hospital. As more residents participate, data collection and analysis will provide greater insight into the simulation’s long-term impact on clinical competence and confidence. By refining the simulation environment and standardizing the debrief process, future iterations aim to further enhance its educational value and reproducibility.

## Conclusions

Quality improvement simulation-based education in anesthesiology residency programs provides a valuable platform for training on high-stakes, complex procedures in a safe and controlled environment. This simulation effectively facilitated the development of essential anesthesiology skills, including endotracheal intubation, epidural catheter placement, and the management of a pregnant patient experiencing hemodynamic instability. Furthermore, it offered learners the opportunity to recognize and manage a high neuraxial blockade - a rare but critical complication of epidural anesthesia - thereby enhancing their preparedness for real-world clinical scenarios.

## Appendices

**Table 4:** Appendix A - High Spinal Stimulation Case Patient age: 21 years; Chief complaint: Painful labor contractions; Physical setting: OBGYN Floor ACLS: Advanced Cardiovascular Life Support; OB/GYN: Obstetrics and Gynecology; IV: Intravenous; ETT: Endotracheal Tube; mm: Millimeter (used to denote the size of the endotracheal tube); OR: Operating Room

Simulation Components	Details/Description
Brief narrative description of the case	The educational goal of performing this simulation is to have a junior learner be able to properly assess a patient for the placement of an epidural catheter, obtain informed consent from the patient, and demonstrate technical proficiency in placing an epidural catheter. For the senior learner, the goal is to create a differential diagnosis for hypotension after epidural placement and ultimately identify and treat a high spinal. Alternatively, this simulation can be performed with a single learner from start to finish.
Primary Learning Objectives	Discuss the risks and benefits of epidural catheter placement and obtain informed consent. Properly assess a patient for the placement of an epidural catheter and ensure proper monitoring is being performed. Demonstrate proper sterile technique and competency in placing an epidural catheter. Identify the signs and symptoms of high neuraxial anesthesia. Demonstrate interdisciplinary collaboration with other specialties. Demonstrate technical ability to perform emergent endotracheal intubation. Analyze critical decision-making in a pregnant, hemodynamically unstable patient with a difficult airway.
Critical Actions	Epidural Catheter Placement- Evaluate the patient prior to the procedure, including a physical exam, airway exam, and review of pertinent labs (blood type & screen, platelet count). Discuss the risks and benefits of epidural anesthesia with the patient and properly obtain informed consent for the procedure. Ensure blood pressure monitoring is set to every three minutes. Describe/demonstrate proper patient positioning for epidural placement. Demonstrate the correct sterile technique for placing the epidural. Assess the patient after placement for the level of blockade or abnormal findings. Management of High Neuraxial Blockade- Recognize early signs of hypotension, bradycardia, and paresthesia, and discontinue epidural infusion. Upon discontinuation of infusion, administer a vasopressor (ephedrine/phenylephrine) and fluid bolus. Request help from the OB/GYN team. Administer a 10-microgram bolus dose of epinephrine to treat refractory hypotension and bradycardia. Sedate and intubate the patient with a 6.5 mm ETT to account for upper airway edema and attach an Ambu bag.
Ideal Scenario Flow	The learners enter the room to find a patient in mild distress with labor contractions. They perform a complete assessment of the patient and appropriately consent her for placement of an epidural catheter. Upon starting the epidural pump, the patient will describe an abnormal sensation in their legs and become hypotensive and bradycardic. The infusion pump should be discontinued, and IV fluids and vasopressors administered. The patient’s cardiovascular status does not improve, and their oxygen saturation begins to decrease. The patient will notify the learners that it is difficult to breathe, and they feel very weak before becoming unresponsive. At this point, the learners should notify the OBGYN team and Anesthesiology team. An epinephrine bolus should be administered, and the learners should prepare to sedate and intubate the patient. Upon intubation, an Ambu bag should be utilized and connected to a source of oxygen while preparing the patient for transport to the OR.
Learner Preparation or Prework	ACLS (Advanced Cardiovascular Life Support), obstetric anesthesia rotation with knowledge/competency in epidural placement.

**Table 5:** Appendix B - Laboratory Results WBC: White Blood Cell count (measured in 10⁹/L); Hgb: Hemoglobin (measured in g/dL); Ptt: Partial Thromboplastin Time (measured in seconds); Lytes: Electrolytes; Na: Sodium (measured in mEq/L); K: Potassium (measured in mEq/L); Cl: Chloride (measured in mEq/L); HCO3: Bicarbonate (measured in mEq/L); AG: Anion Gap; BUN: Blood Urea Nitrogen (measured in mg/dL); Cr: Creatinine (measured in mg/dL); Glucose: Blood sugar level (measured in mg/dL); B-HCG: Beta-Human Chorionic Gonadotropin.

Laboratory Test	Result (Reference Range)
WBC 9.86	4.5-11
Hgb 11.5	12.1-15.1
Ptt 112	25-35
-	-
Lytes	-
Na 136	136-144
K 3.7	3.7-5.1
Cl 105	97-105
HCO3 20	22-30
AG 15	Oct-18
BUN 7	Jun-24
Cr 0.69	0.6-1.1
Glucose 104	70-100
-	-
Other	-
B-HCG Positive	-

**Table 6:** Appendix C - Critical Actions Checklist q3 min: Every 3 minutes; OB/GYN: Obstetrics and Gynecology; mcg: Micrograms; ETT: Endotracheal Tube; mm: Millimeter

1. Epidural catheter placement
□ Evaluates the patient prior to procedure including physical exam, airway exam, and review of pertinent labs (blood type & screen, platelet count).
□ Discusses the risks and benefits of epidural anesthesia with the patient and properly obtains informed consent for the procedure.
□ Checks that blood pressure monitoring is set to q3 min.
□ Describes/demonstrates proper patient positioning for epidural placement.
□ Demonstrates correct sterile technique in placing epidural.
□ Assesses the patient after placement for level of blockade or abnormal findings.
2. Management of high neuraxial blockade
□ Recognizes early signs of hypotension, bradycardia and paresthesia and discontinues epidural infusion.
□ Upon discontinuation of infusion, administers vasopressor (ephedrine/phenylephrine) and fluid bolus.
□ Requests help from OB/GYN team
□ Administers epinephrine 10 mcg bolus dose to treat refractory hypotension and bradycardia.
□ Sedate and intubate patient with 6.5 mm ETT to account for upper airway edema and attach Ambu bag.

**Table 7:** Appendix D - Debriefing information ASA: American Society of Anesthesiologists; CSF: Cerebrospinal Fluid; B-HCG: Beta-Human Chorionic Gonadotropin; NPO: "Nil Per Os" (nothing by mouth); RSI: Rapid Sequence Induction

Debriefing Topic	Key Discussion Points
1. Steps for informed consent	Obtaining informed consent from a parturient for the placement and administration of epidural anesthesia is an essential task of an anesthesiologist. It is important that the patient be made aware of the benefits, risks, and side effects associated with the procedure, in addition to an easily comprehended explanation of the process. After such a conversation with the patient and with their understanding and agreement, the anesthesiologist must ensure proper documentation of the informed consent process, including the signatures of themselves, the patient or their appropriate representative, and a witness.
2. Importance of reviewing labs prior to epidural	As part of the preanesthetic evaluation, a focused history and physical exam should be performed, and the patient’s back should be examined for the placement of neuraxial anesthesia. Any pertinent labs should be reviewed, such as blood type and screen, with an emphasis on coagulopathy indicators, while considering any medications the patient may currently be taking, such as heparin [12]. According to the Practice Guidelines for Obstetric Anesthesia [3], “the anesthesiologist’s decision to order or require a platelet count should be individualized and based on a patient’s history (e.g., preeclampsia with severe features), physical examination, and clinical signs.”
3. Steps for epidural placement	Prior to the procedure, standard ASA monitoring must be in place. Discuss proper positioning and identification of the target level on the patient in both the sitting and lateral decubitus positions. The administering anesthesiologist should practice sterile technique while performing this procedure. The steps of the procedure should be demonstrated/discussed, including: application of local anesthetic, introduction of the epidural needle through the supraspinous and interspinous ligaments, application of a glass syringe filled with air or normal saline on the epidural needle, advancement of the epidural needle through the ligamentum flavum, injecting the glass syringe for loss of resistance, catheter placement, aspiration of the catheter to ensure no CSF return, administration of a test dose of 3 mL of 1.5% lidocaine with 1:200,000 epinephrine, placement of a sterile dressing, and securing the catheter with tape. [12]
4. Etiology of high spinal/high neuraxial block	A high neuraxial block or “high spinal” is the spread of local anesthetic to an unintended vertebral level. Unintentional intrathecal injection of large doses of local anesthetic can cause a high spinal [1]. If the cranial nerves are affected, then the neuraxial blockade is known as a “total spinal.”
5. Signs and symptoms	The symptoms of high neuraxial blockade include bradycardia, hypotension, and respiratory depression. The initial sign of hypotension may present as nausea, while other initial signs of a high spinal may be numbness/weakness in the UE, dyspnea, and difficulty with phonation.
6. Treatment	Quickly recognizing the signs and symptoms of high neuraxial blockade and treating them appropriately is critical to patient outcomes. Treatment includes maintaining arterial oxygenation (supplemental O2), supporting the airway as needed (intubate and place on mechanical ventilation), treating bradycardia (atropine), and hypotension (IV fluids + ephedrine, phenylephrine, or epinephrine).
7. Sedation medications for intubation in a parturient patient	Midazolam may be utilized prior to intubation for anxiolysis in the parturient patient. In one double-blind, randomized, placebo-controlled trial involving 60 healthy pregnant women, sedation with fentanyl (1 microgram per kg) and midazolam (0.02 mg per kg) immediately prior to spinal anesthesia was not associated with adverse neonatal effects. Propofol and etomidate are commonly used as sedatives for intubation in healthy parturient patients. Ketamine may also be considered in hypovolemic patients. For most pregnant patients, adjunctive medications such as opioids are avoided until delivery of the fetus to avoid potential newborn complications [13][14].
8. Should parturient patients experiencing a high spinal receive sedation?	To safely intubate the parturient patient experiencing a high spinal so that emergent cesarean section delivery can be performed under general anesthesia, it is typically necessary to provide sedation. In a hemodynamically unstable patient, it is important to consider the choice of induction medication. In a parturient patient undergoing emergent cesarean section delivery due to a high neuraxial blockade from epidural anesthesia, hypotension is the major symptom to consider when choosing an induction agent. Propofol should be avoided due to its blood pressure-lowering effect. Etomidate could be used as it is a more hemodynamically stable choice for induction [15]. However, ketamine may be considered a first-line induction agent for parturient patients experiencing high neuraxial blockade as “the sympathomimetic properties of ketamine make it an ideal induction agent in the setting of emergent cesarean delivery in a patient with hypotension…” [16]. Opioids should be avoided in this patient until after delivery of the fetus due to medication transfer as well as exacerbating the respiratory depression of the mother.
9. Concerns of intubating a pregnant patient	The physiologic changes associated with pregnancy can make intubation more challenging. Airway edema, decreased functional residual capacity, and weight gain are just a few of the changes that have been shown to contribute to an increase in the number of Class IV Mallampati scores in pregnant patients [17]. Regardless of a pregnant patient’s NPO status, “all obstetric patients are considered to have a full stomach and to be at risk for pulmonary aspiration.” As such, rapid sequence induction should be utilized in pregnant patients to reduce the risk of aspiration. Additionally, administration of metoclopramide and/or an H2 receptor antagonist should be considered to reduce the risk for aspiration pneumonitis [18].
10. Difficult airway algorithm for pregnant patients	Review the ASA Difficult Airway Algorithm [19] for adult patients as it pertains to a parturient undergoing an emergent cesarean section.
11. Effective communication with OBGYN team	Communication between the patient, OBGYN team, and the anesthesiologist is critical to achieving good patient outcomes. Effective communication begins with the informed consent process between the anesthesiologist and the patient for the epidural anesthesia, as well as ensuring that the OBGYN team is aware of that plan and ready to perform an emergent cesarean section if need be. Intraoperatively, the anesthesiologist needs to communicate with the OBGYN team about important steps in their management of the patient’s airway as well as upon administration of various medications. Oxygen desaturation should trigger appropriate actions from learners, including calling for the OBGYN team, administering epinephrine, and preparing for intubation of the patient.

## References

[1] Hawkins JL (2010). Epidural analgesia for labor and delivery. N Engl J Med.

[2] D'Angelo R, Smiley RM, Riley ET, Segal S (2014). Serious complications related to obstetric anesthesia: the serious complication repository project of the Society for Obstetric Anesthesia and Perinatology. Anesthesiology.

[3] (2016). Practice guidelines for obstetric anesthesia: an updated report by the American Society of Anesthesiologists Task Force on Obstetric Anesthesia and the Society for Obstetric Anesthesia and Perinatology. Anesthesiology.

[4] (2020). ACGME Program Requirements for Graduate Medical Education in Anesthesiology. The Accreditation Council for Graduate Medical Education website. https://www.acgme.org/globalassets/pfassets/programrequirements/040_anesthesiology_2021.pdf.

[5] Accreditation Council for Graduate Medical Education (2021). Anesthesiology Milestones. https://www.acgme.org/Portals/0/PDFs/Milestones/AnesthesiologyMilestones.pdf.

[6] Accreditation Council for Graduate Medical Education (2022). Obstetric Anesthesiology Milestones. https://www.acgme.org/globalassets/pdfs/milestones/obstetricanesthesiologymilestones2.0.pdf.

[7] Aird D, Ellis T, Krishnan S (2016). Problem-based learning discussion on neuraxial anesthesia in parturients with repaired tetralogy of Fallot. MedEdPORTAL.

[8] Lee A, Sheen JJ, Richards S (2018). Intrapartum maternal cardiac arrest: a simulation case for multidisciplinary providers. MedEdPORTAL.

[9] Kariya N, Kawasaki Y, Okutani H, Kaneko T, Ueki R, Hirose M (2020). Effects of simulation study of high neuraxial block during epidural analgesia for labor pain on pre/posttest evaluation in junior clinical trainees. Anesth Pain Med.

[10] Eppich W, Cheng A (2015). Promoting Excellence and Reflective Learning in Simulation (PEARLS): development and rationale for a blended approach to health care simulation debriefing. Simul Healthc.

[11] (2025). Guidelines for Neuraxial Analgesia or Anesthesia in Obstetrics. Anesthesia in Obstetrics.

[12] Holladay J (2025 Jan-). Epidural catheter. StatPearls [Internet].

[13] Frölich MA, Burchfield DJ, Euliano TY, Caton D (2006). A single dose of fentanyl and midazolam prior to Cesarean section have no adverse neonatal effects. Can J Anaesth.

[14] Nixon H, Leffert L (2022). Anesthesia for cesarean section. UpToDate.

[15] Frank RL (2022). Procedural sedation in adults outside the operating room. UpToDate.

[16] Chestnut DH, Tsen Laurence C, Bateman Brian T (2020). 26. Anesthesia for cesarean delivery. Chestnut's Obstetric Anesthesia: Principles and Practice, 6th Edition.

[17] Chestnut DH, Russell R (2020). 29. The difficult airway: risk, assessment, prophylaxis, and management. Chestnut's Obstetric Anesthesia: Principles and Practice. 6th ed. Elsevier.

[18] Frölich MA (2022). Obstetric anesthesia. Morgan & Mikhail’s Clinical Anesthesiology, 7e.

[19] Apfelbaum JL, Hagberg CA, Connis RT (2022). 2022 American Society of Anesthesiologists Practice Guidelines for Management of the Difficult Airway. Anesthesiology.

